# Use of a Potential Probiotic, *Lactobacillus casei* L4, in the Preparation of Fermented Coconut Water Beverage

**DOI:** 10.3389/fmicb.2018.01976

**Published:** 2018-08-22

**Authors:** Sib S. Giri, Venkatachalam Sukumaran, Shib S. Sen, Se Chang Park

**Affiliations:** ^1^Department of Biotechnology, Periyar Maniammai University, Thanjavur, India; ^2^Laboratory of Aquatic Biomedicine, College of Veterinary Medicine and Research Institute for Veterinary Science, Seoul National University, Seoul, South Korea; ^3^School of Life Sciences, Jawaharlal Nehru University, New Delhi, India

**Keywords:** coconut water, anti-oxidant activity, mineral, *Lactobacillus*, functional beverage, vitamin

## Abstract

Coconut water (CW) is a clear, nutritive liquid found as the coconut endosperm of green coconuts such as *Cocos nucifera* L., and its widespread consumption owes to its unique composition of sugars, minerals, vitamins, enzymes, and hormones. Probiotic fermentation of CW may facilitate the development of an improved functional beverage with probiotic benefits; therefore, we aimed to produce a fermented CW beverage using the potential probiotic *Lactobacillus casei* L4. CW was fermented with *L. casei* L4 for 48 h at 35°C, and the pH, organic acid-production rate, antioxidant activity, antibacterial activity, sugar, mineral, vitamin B12 levels, and total viable bacteria counts were investigated at 24 and 48 h. We demonstrated that the fermentation of CW with probiotic lactobacilli increased the cell viability count. Vitamin B12 production was highest in the extracellular environment at 48 h (11.47 μg/mL), while the total phenolic content was significantly (*p* < 0.05) higher in the fermented CW at 48 h (72.1 μg/mL gallic acid equivalents) than observed with the other investigated groups or time points. The fermented materials exhibited the highest 1,1-diphenyl-2-picrylhydrazyl (DPPH) and 2,2′-azino-bis(3-ethylbenzothiazoline-6-sulfonic acid) (ABTS) radical-scavenging activities at 48 h (58.4 and 69.2%, respectively). The levels of most minerals remained unchanged in the fermented CW, except for calcium, manganese, phosphorus, and sodium. Furthermore, the culture supernatant from fermented CW inhibited the growth of foodborne pathogens such as *Bacillus cereus*, *Listeria monocytogenes*, *Staphylococcus aureus*, and *Salmonella typhi*, although the degree of inhibition varied between the species. Moreover, adding 15% honey and artificial coconut flavor to the fermented CW resulted in a better-tasting product, as demonstrated by a sensory-evaluation test. The obtained results indicated that the CW product fermented by *L. casei* L4 may be used as a novel functional beverage containing both electrolytes and probiotics, and can serve as a good vehicle for preparing a wider range of novel products.

## Introduction

The coconut *Cocos nucifera* L. belongs to the Arecaceae (Palmae) family and is an important member of the monocotyledons, which are grown throughout tropical and sub-tropical regions (Manivannan et al., 2018). Coconut water (CW) is the clear, nutritive liquid obtained from the endosperm of coconuts. CW is classified as tender CW (TCW) or matured CW (MCW), based on the harvest time (Zhang et al., 2018). Generally, in the tropics, TCW is consumed primarily as a sports drink (Cappelletti et al., 2015), whereas MCW is generally discarded, because only coconut meat is used for various culinary purposes. CW is low in calories and fat, but rich in sugars, vitamins, amino acids, and minerals; therefore, it represents a natural alternative to artificial sports drinks for replenishing electrolytes following exercise (Saat et al., 2002; Yong et al., 2009; Seesuriyachan et al., 2011). Edible coconut parts and various value-added products contain high levels of phenolic and flavonoid compounds with antioxidant potential (Arivalagan et al., 2018), and (+)-catechin and (−)-epicatechin were reported in CW as well (Chang and Wu, 2011). DebMandal and Mandal (2011) reviewed the antioxidant, cardioprotective, antithrombotic, hypolipidemic, anti-cholecystitis, antibacterial, antiviral, antifungal, antiprotozoal, immunostimulatory, antidiabetic, and hepatoprotective properties of coconuts. However, the use of CW to prepare fermented beverages has only been rarely reported (Lee et al., 2013; Prado et al., 2015; Kantachote et al., 2017; Zhang et al., 2018).

The functional food field has developed in recent years. Functional beverages are defined as non-alcoholic drinks reinforced with ingredients such as herbs, vitamins, minerals, amino acids, or probiotics, and provide specific health benefits beyond those in other common food sources (Corbo et al., 2014). The regular consumption of functional foods has beneficial effects on health, such as antioxidant or antiatherogenic effects (Cirillo et al., 2016). Further, drinks formulated specifically for rehydration should be able to replace water lost through sweating and replenish electrolytes, especially sodium (Saat et al., 2002).

Probiotic foods were previously estimated to comprise approximately 60–70% of the total functional food market (Tripathi and Giri, 2014). Probiotics are defined as “live microorganisms that, when administered in adequate amounts, confer a health benefit to the host” (Hill et al., 2014). Probiotic bacteria are used as nutritional supplements in functional foods, which are easily digestible. Lactic acid bacteria (LAB) are most commonly used for these purposes (Plessas et al., 2017), and of these, members of the *Lactobacillus* and *Bifidobacterium* genera are predominantly associated with food fermentation (Plessas et al., 2017; Giri et al., 2018). LAB and other starter cultures transform the biochemical and organoleptic characteristics of substrates, produce various metabolites, and enrich beverages/foods with micronutrients such as minerals, vitamins, amino acids, probiotics, prebiotics, and digestive enzymes (Ray et al., 2016). The health-improving properties of probiotic LAB are associated with their positive influences on the intestinal microbiota, intestinal functions, inhibition of pathogenic microorganism growth, production of B vitamins (especially folic acid), immune response stimulation, and the reduction of lactose-intolerance symptoms (Enujiugha and Badejo, 2017). In addition to their use in the preparation of various fermented dairy and non-dairy products, *Lactobacillus* species have been successfully used to prepare fermented CW beverages (Lee et al., 2013; Prado et al., 2015; Watawana et al., 2016; Kantachote et al., 2017). Therefore, new strains of lactobacilli having interesting functional traits, isolated from traditional fermented products, may be useful for probiotic applications. Fermentation of CW with probiotic LAB can produce novel probiotic beverage which can provide hydration and probiotic benefits to individuals.

The quantification of organic acids, including lactic, acetic, malic acid, among others, in the fermented product is important from technical, nutritional, sensorial, and microbial-content standpoints (Shukla et al., 2010). Some LAB strains were shown to produce cholesterol-lowering, antimicrobial, antioxidant, immune-modulating, chaperone-like, and opioid/opioid antagonist peptides (Enrica and Simona, 2016). Vitamin B12 (cobalamin) deficiency was previously associated with hematological and neurological disorders and myocardial infarction, as humans cannot produce vitamin B12, but instead obtain it primarily through foods of animal origin, such as milk, meat, and eggs (Pawlak, 2015). Therefore, vegetarians have an increased risk of developing vitamin B12 deficiency. However, only a few archaeal and bacterial species can produce large quantities of vitamin B12, such as *Lactobacillus plantarum* LZ95 (Li et al., 2017) and *Lactobacillus reuteri* CRL1098 (Taranto et al., 2003). Previously, we isolated *Lactobacillus casei* L4 from the Indian ethnic fermented rice beverage “bhaati jaaner” (Giri et al., 2018) and demonstrated that it exhibits satisfactory *in vitro* probiotic properties, including acid and bile tolerance, antibiotic susceptibility, antagonistic activity against foodborne pathogens, cell surface hydrophobicity, and auto-aggregation. Therefore, in this study, we focused on the production of a functional beverage from CW using *L. casei* L4, with the aim of improving the health benefits of this beverage. Additionally, we analyzed alterations in the antioxidant activity, organic acid production, sugar content, mineral accumulation, vitamin B12 production, and antibacterial activity of the fermented CW.

## Materials and Methods

### Starter Culture

The *L. casei* L4 strain was previously isolated from the ethnic fermented rice beverage bhaati jaaner and shown to have probiotic properties *in vitro* (Giri et al., 2018). The L4 isolate was identified based on its biochemical and morphological properties, and the phenotypic identification was confirmed by *16S rRNA* gene sequencing, which revealed 99% similarity to the corresponding gene sequence of several *L. casei* strains. The partial *16S rRNA* gene sequence of the L4 isolate was deposited in GenBank under accession number MH298846.

The culture was propagated in sterile de Man Rogosa Sharpe (MRS) broth (Sigma-Aldrich, St. Louis, MO, United States) for 24 h at 35°C. Stocks were prepared routinely in the MRS medium. To prepare the starter culture, the broth culture was centrifuged at 8000 × *g* for 15 min, and the pellet was washed twice with 0.85% NaCl. Subsequently, the pellets were suspended in sterile CW (see below), the cell count was measured, and this suspension was used as a starter culture.

### Coconut Water Fermentation

Fresh green coconuts (approximately 5 months old) were obtained from a local market in Thanjavur, Tamil Nadu, India. The CW was obtained by perforating the coconut with a sterile knife after the primary epicarp was brushed and washed with distilled water. The water from 30 green coconuts was collected. The initial total solid content (°Brix) was 6.41% and the pH was 6.49. The CW was pre-filtered through a 0.65-μm polyethersulphone filter (Sigma-Aldrich) before being filtered through 0.45-μm filter paper (Millipore, United States). The pH of filtered CW was adjusted to 6.5 to achieve the optimum pH for the growth of *L. casei* L4 in MRS medium (Giri et al., 2018). Filtered CW (1 L) was inoculated with a 1.0% (v/v) pure culture of *L. casei* L4 at a final concentration of 10^8^ cells/mL. The batch fermentations were performed statically in triplicate in three conical flasks (2 L) for 48 h at 35°C. Samples were collected aseptically at 0, 24, and 48 h, and immediately stored at −80°C. During a preliminary 96-h fermentation study, the fermented product was collected at 24-h intervals and tested for viable counts, antioxidant activity, and total acidity. Based on the results of the preliminary study, 48 h was selected as the fermentation time.

Supernatants were obtained from the culture samples after centrifugation at 8000 × *g* for 10 min at 4°C. The culture broth and biomass were used to determine several parameters.

### Determination of pH, Total Acidity, and Cell Viability

The pH was determined using a digital pH meter (Sigma-Aldrich, United States). The total titratable acidity was measured by homogenizing 10 g of each sample with 90 mL distilled water, followed by titration with 0.1 N NaOH using 0.1% (w/v) phenolphthalein in 95% ethanol as an indicator. Samples collected at 0 h were used as a control.

Lactic acid bacteria viable counts on MRS agar plates were obtained for the fermented products. One milliliter of each sample was diluted with 0.1% (w/v) bacteriological peptone (Sigma-Aldrich), and the diluted samples (0.1 mL) were spread on triplicate MRS agar plates. Viable LAB counts were determined up through 2 days of storage at room temperature, and then every week for 4 weeks after storage at 4°C. The viability was recorded as the number of colony-forming units (CFU) per mL, and the cell concentration was expressed as log of the CFU/mL.

### Antibacterial Activity of Fermented CW

The antibacterial activities of the culture supernatants were determined using the agar-diffusion method (Garcia et al., 2016). Culture supernatants were filtered through a 0.45-μM Millipore filter to obtain cell-free supernatants. The following foodborne pathogens were studied as target organisms: *Bacillus cereus* MTCC6629, *Listeria monocytogenes* MTCC1143, *Staphylococcus aureus* MTCC737, and *Salmonella typhi* ATCC19430. Briefly, each strain was aerobically grown overnight in brain heart infusion (BHI; Sigma-Aldrich) broth at 37°C and centrifuged (4500 × *g*, 15 min, 4°C), after which the pellets were washed and resuspended in sterile saline solution. A 1-mL aliquot of each bacterial suspension was spread onto BHI soft agar plates (final viable counts of approximately 8 log CFUs/mL), and 50 μL of each cell-free supernatant was dispensed into wells containing BHI agar (5-mm diameter and 5-mm depth; drilled using a sterile glass cannulas). The plates were aerobically incubated at 37°C for 48 h, and the diameter (mm) of the growth-inhibition zones around each well was measured. MRS broth was used as the negative control, and un-inoculated CW was used as the positive control.

### Analytical Determination

Sugars and organic acids were quantified by high-performance liquid chromatography as previously described (Miguel et al., 2012; Plessas et al., 2017) and identified according to the retention time and standard curves of the corresponding standards (Sigma-Aldrich). Samples collected at 0 h were used as controls.

To determine mineral contents, a 5-g sample collected at the end of the fermentation was homogenized in 25 mL deionized distilled water and centrifuged at 12,000 × *g* for 10 min to collect the supernatant. The concentration of minerals in the supernatant was then determined by atomic absorption spectrophotometry (PerkinElmer, United States) and flame photometry methods (AOAC, 2005). Un-inoculated CW was used as a control.

Total phenolic contents were determined according to the Folin–Ciocalteu colorimetric method described by de Camargo et al. (2012). A calibration curve was obtained using gallic acid (0–8.2 μg/mL) as the standard. Results were expressed as the micrograms of gallic acid equivalents (GAEs) per mL of sample. The 1,1-diphenyl-2-picrylhydrazyl (DPPH) and 2,2′-azino-bis(3-ethylbenzothiazoline-6-sulfonic acid) (ABTS) radical-scavenging activities (RSAs) was determined using the modified method of Freire et al. (2017). RSAs were calculated as follows: RSA (%) = (1–A_sample_/A_control_) × 100, where A_control_ is the absorbance of the blank sample, and A_sample_ is the absorbance of the investigated sample. Samples collected at 0 h were used as controls.

Vitamin B12 contents in the culture supernatants and biomass were determined to measure the intracellular contents and extracellular contents, respectively, following the method described by Kantachote et al. (2017).

### Sensory Evaluation

Sensory evaluation of the fermented coconut beverages was performed after 28 days of storage at 4°C by a group of 20 panelists (age group: 21–60 years old) using the Hedonic 5-point scale. The participants evaluated the beverages based on the color, appearance, flavor, texture, and overall acceptance. Three variations of this beverage were provided to panelists: fermented CW, fermented CW supplemented with 15% honey, and fermented CW supplemented with 15% (v/v) honey and 0.5 % (w/v) coconut flavor (Apex Flavors Inc., Belcamp, MD, United States; ingredients: water, ethyl alcohol, propylene glycol, and natural flavor).

### Statistical Analysis

The obtained data were analyzed by one-way analysis of variance, and Tukey’s test was employed to assess differences between treatments. All statistical analyses were performed using OriginPro software (version 8; OriginLab Corporation, Northampton, MA, United States). *p* < 0.05 was considered statistically significant, and the results are expressed as mean values ± standard deviations.

## Results and Discussion

### Probiotic Bacterial-Growth Profile During Fermentation

*Lactobacillus casei* L4 showed a good growth rate during the fermentation period (**Table 1**). The cell count significantly increased at 24 h, compared with that in the 0 h (cell viability, 9.31 log CFU/mL; *p* < 0.05) and thereafter further increased slightly to 9.47 log CFU/mL at 48 h. This cell growth was accompanied by a significant increase (*p* < 0.05) in total acidity, which was 0.77% after 48 h when the pH dropped to 3.32. High viability counts are necessary to obtain the desired levels of acid production and pH reduction, which affects the organoleptic properties of the product. Further, the bacterial cells survived at a count of 7.4 log CFU/mL after a 1-week incubation at room temperature (25–33°C), while, after a 28-day storage at 4°C, the cell count was 7.84 log CFUs/mL. Similarly, CW fermented with *Lactobacillus acidophilus* and *L. casei* had considerably high viability, with cell counts of 5.04 × 10^7^ and 1.80 × 10^8^CFUs/mL, respectively, after 28 days of storage at 4°C (Lee et al., 2013). The cell concentrations in the fermented CW after 48 h were above the minimum recommended for a probiotic product (6 log CFUs/mL based on a daily dose of 100 mL) (Sanders and Huis In’t Veld, 1999). The final count of the *Lactobacillus* cells was comparable to those previously reported in MCW after 48 of fermentation with *L. plantarum* DW12 (Kantachote et al., 2017) and young CW fermented with *L. acidophilus* L10 and *L. casei* L26 (Lee et al., 2013).

**Table 1:** Cell viability in coconut water fermented with *L. casei* L4.

Parameter	Fermentation time (h)	CW
Viable cells (Log CFU/mL)	0	8.02 ± 0.05^a^
	24	9.31 ± 0.06^bc^
	48	9.47 ± 0.03^b^
	Days of storage	
	7 (at room temp.)	7.4 ± 0.04
	7 (at 4°C)	9.32 ± 0.08
	14 (at 4°C)	9.03 ± 0.12
	21 (at 4°C)	8.58 ± 0.06
	28 (at 4°C)	7.84 ± 0.07
pH	0	6.5 ± 0.01^a^
	24	4.02 ± 0.04^b^
	48	3.32 ± 0.05^c^
Total acidity (%)	0	0.05 ± 0.00
	24	0.61 ± 0.03
	48	0.77 ± 0.02

*Results are presented as mean ± SD (n = 3). Values in the column (for pH or viable cells) with different superscript letters are are significantly different.*

### Antibacterial Activity

The activities of the bacterial culture supernatants against selected foodborne pathogens are presented in **Table 2**. The obtained results demonstrated that the growth of all investigated pathogens was inhibited. The highest activity was observed against *L. monocytogenes*, followed by that against *S. typhi*, *S. aureus*, and finally *B. cereus.* The sensitivity of *L. monocytogenes* was similar to that reported in a recent study, which showed that fermented MCW inhibited the growth of *L. monocytogenes* (Kantachote et al., 2017), and those authors showed a much greater zone of inhibition. However, in our earlier study, we have shown the antagonistic activity of the neutralized culture supernatant of L4 (identified as *L. casei*) against foodborne pathogens (Giri et al., 2018). In that study, the inhibition zones against pathogens (*S. aureus* MTCC737, *L. monocytogenes* MTCC1143, and *B. cereus* MTCC6629) ranged between >2 mm and >5 mm (Giri et al., 2018), which are comparable to those observed in this study. The production of various organic acids (described below) and other bioactive substances in the fermented product may be involved in inhibiting pathogen growth. The antimicrobial properties of the fermented product suggest that it can be safely preserved for human consumption.

**Table 2:** Antibacterial activity of fermented coconut water product against foodborne pathogens.

Target organisms	Zone of inhibition (mm)	
	24 h	48 h
*Bacillus cereus* MTCC6629	2.8 ± 0.07	3.1 ± 0.11
*Listeria monocytogenes* MTCC1143	4.2 ± 0.33	5.1 ± 0.24
*Staphylococcus aureus* MTCC737	2.9 ± 0.08	3.4 ± 0.16
*Salmonella typhi* ATCC19430	3.6 ± 0.12	3.8 ± 0.13

### Antioxidant Activity

**FIGURE 1:**
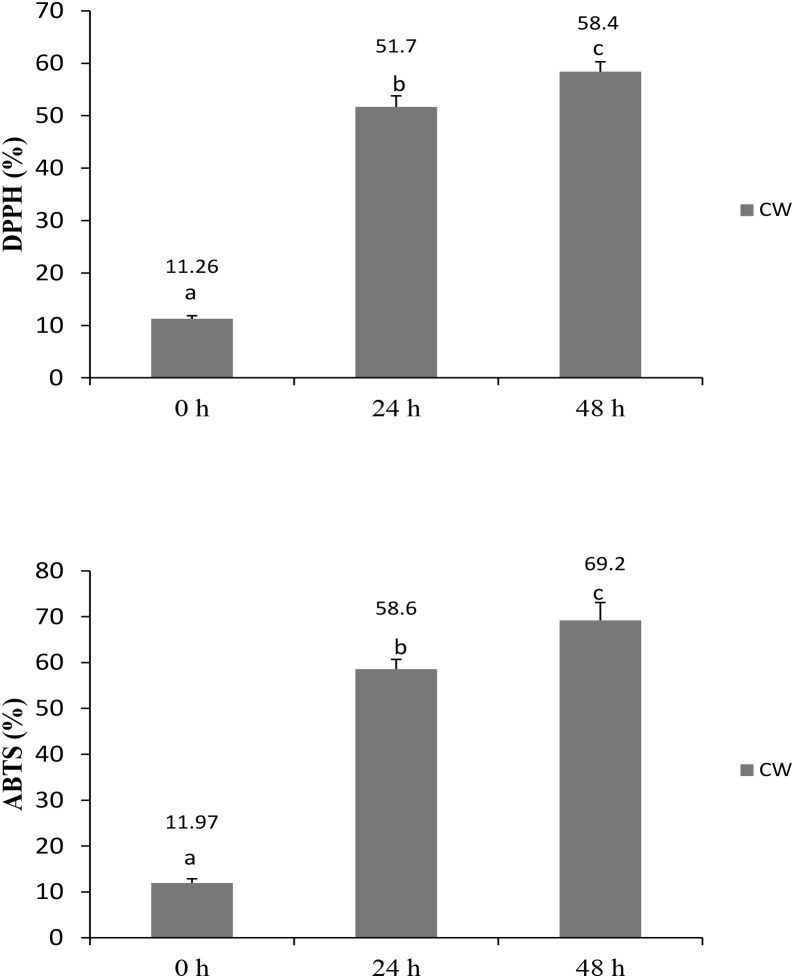
Radical scavenging activities of CW fermented with *L. casei* L4 as measured by DPPH and ABTS assays. Different letter above the bar indicate significant different (*p* < 0.05).

Antioxidant foods and beverages have become increasingly popular because of their potential health benefits, which are attributed mostly to their bioactive compounds (Abountiolas and Nascimento Nunes, 2018). No standardized method has been established to determine the antioxidative abilities of food and beverages; therefore, the application of two or more methods is recommended to determine the antioxidative levels in a sample. In this study, RSAs of fermented CW was evaluated by performing DPPH and ABTS radical-scavenging assays, and total phenolic contents were determined as well. The RSA increased significantly in the fermented culture supernatants at both 24 and 48 h, compared with that measured at 0 h. DPPH and ABTS radical-scavenging activities in the fermented culture supernatants at 48 h were 58.4 and 69.2%, respectively (**Figure 1**), showing that the *L. casei* L4 culture had antioxidant activity. These findings are in agreement with the results of Kantachote et al. (2017), who reported that the RSA of CW fermented using *L. plantarum* DW12 at 48 h was 73.4% for ABTS and 54.87% for DPPH. Recently, Zhang et al. (2018) demonstrated an increase in antioxidative activities during a 48-h CW fermentation by *Saccharomyces cerevisiae*.

**FIGURE 2:**
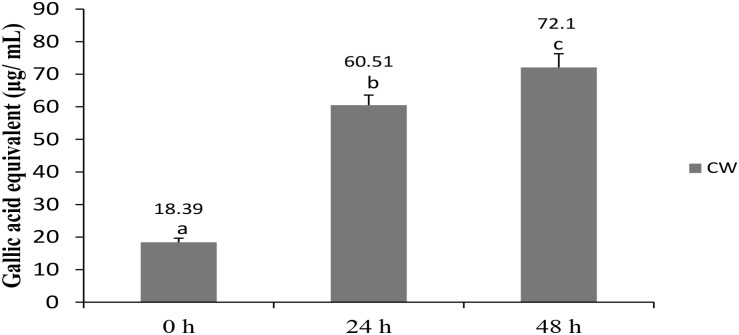
Total phenolic content in CW fermented by *L. casei* L4. Results are expressed as mean ± SD (*n* = 3). Different letter above the bar indicate significant difference (*p* < 0.05).

The total phenolic content of fermented CW increased significantly from 18.39 μg/mL GAEs at 0 h to 60.51 μg/mL GAEs at 24 h and further to 72.1 μg/mL GAEs at 48 h (**Figure 2**). Phenolic compounds, widely distributed in plants, are the most abundant antioxidants in the human diet (Giri et al., 2018). Some of them, including (+)-catechin and (−)-epicatechin, were detected in CW (Chang and Wu, 2011), whereas other researchers reported an increase in the total phenolic content in cereal-based beverages following fermentation with probiotic lactobacilli (Ghosh et al., 2015; Freire et al., 2017; Giri et al., 2018). An increase in the total phenolic content in CW fermented with lactobacilli and/or yeast, in comparison with that in non-fermented CW, was observed in several studies (Watawana et al., 2016; Kantachote et al., 2017; Zhang et al., 2018). However, presence of compounds rather than total phenolic contents may also be responsible for antioxidant properties as suggested by earlier researcher who used *L.* *plantarum* DW12 in fermenting CW (Kantachote et al., 2017).

### Sugar and Organic Acid Production

Glucose and fructose were detected in CW (**Table 3**), and significant changes in the sugar composition were observed after 24 h of fermentation, compared with that found after 0 h. The glucose and fructose levels in fermented CW were 26.7 and 25.1 g/L, respectively, after 48 h of fermentation (**Table 3**). Similarly, significant reductions in sugar levels in CW fermented with *L. plantarum* AC-1 (Prado et al., 2015) and CW fermented with *L.* *casei* L26 and/or *L. acidophilus* L10 (Lee et al., 2013) were previously observed.

**Table 3:** Concentration of sugars in coconut water fermented with *L. casei* L4.

Fermentation time	Glucose (g/L)	Fructose (g/L)
0 h	48.3^a^	37.4^a^
24 h	33.4^b^	29.8^b^
48 h	26.7^c^	25.1^c^

*Results are presented as mean ± SD (n = 3). Values in a column with unlike superscript letters are significantly different.*

Organic acid levels and compositions are important indicators of the metabolic processes occurring during the storage of a fermented product (De Dea Lindner et al., 2013). In this study, the pH, glucose, and fructose levels decreased, and lactic acid production increased during fermentation, as expected. *L. casei* L4-associated fermentation of CW caused alterations in organic acid production (**Table 4**) as well. The levels of the main organic acids of raw CW (such as oxalic, citric, and malic acid) decreased, but that of tartaric acid increased in fermented CW (**Table 4**). Among the organic acids, the levels of lactic acid increased most prominently to 6.8 and 7.9 g/L after 24 and 48 h, respectively. *L. casei* is homofermentative and, therefore, produced lactic acid as major organic acid. Similar results were reported by Prado et al. (2015). Similar trends in the production of tartaric acid, oxalic acid, and citric acid have been observed during a 48-h fermentation of CW by *S. cerevisiae* (Zhang et al., 2018). However, significantly lower production of acetic acid was also reported by the same authors, which contrasts with the results of this study. During fermentation, the malic acid content was relatively stable, while the acetic acid concentration increased. These findings may reflect the dynamic balance between consumption and generation in the tricarboxylic acid cycle (Zhang et al., 2018).

**Table 4:** Production of organic acids by the action of *Lactobacillus casei* L4 during the course of fermentation.

Time	Organic acid productions					
	Lactic acid (g/L)	Acetic acid (g/L)	Malic acid (g/L)	Tartaric acid (g/L)	Oxalic acid (mg/L)	Citric acid (g/L)
0 h	0	0	0.21^a^	0.02^a^	54.6	0.17^a^
24 h	6.8^a^	0.13^a^	0.16^b^	0.13^b^	ND	0.03^b^
48 h	7.9^b^	0.18^b^	0.14^b^	0.15^b^	ND	ND

*Results are presented as mean ± SD (n = 3). Values in a column with unlike superscript letters are significantly different.*

Moreover, despite the increase in lactic acid production, a substantial amount of residual sugars was detected in the fermented broth after 2 days of fermentation. Similarly, CW fermented with *L. casei* L26 or *L. acidophilus* L10 retained >50% of their residual sugar contents after 48 h fermentation (Lee et al., 2013). In a similar study, Prado et al. (2015) reported the presence of >70% residual glucose and fructose after 10 h of fermentation. Mousavi et al. (2011) reported that the *Lactobacillus-*associated metabolism of carbohydrates varies across strains and depends on the substrate and the duration of fermentation. Those authors found high levels of residual sugars in pomegranate juice even after 3 days of fermentation by *Lactobacillus* strains. The presence of high levels of residual glucose and fructose can assist in the continuous metabolic activity of the strains and generate post-acidification in the fermented product (Prado et al., 2015). Further, high concentrations of residual sugar would enable the retention of sweetness.

The observed slight decrease in malic production agrees with previous results (Lee et al., 2013) demonstrating that most LAB can decarboxylate malate to lactate and CO_2_ through the malolactic enzyme, although *L. casei* may degrade malate in a different manner: malate → pyruvate + CO_2_ → lactate (Landete et al., 2010). Citric and malic acid were reported to protect the myocardium and prevent ischemic lesions (Tang et al., 2013), and organic acids are crucial for promoting the flavor, taste, and color of the product (Esteves et al., 2004). Moreover, lactic acid in the fermented products contributes to the mildly sour taste (Giri et al., 2018).

### Mineral Content and Vitamin B12 Production

Minerals are essential inorganic elements necessary for the regulation of metabolic processes and structural functions (Frazier, 2009). Calcium, magnesium, potassium, phosphorus, and sodium were detected as major mineral components of both fermented and unfermented CW (**Table 5**), consistent with previous reports (Lee et al., 2013; Kantachote et al., 2017). Magnesium is a co-factor of many enzymes, involved in protein synthesis and energy metabolism (Berdanier and Berdanier, 2015). In this study, most of the mineral levels remained relatively unchanged. However, calcium and sodium levels increased (*p* < 0.05) in the fermented CW compared with those in unfermented fresh CW (**Table 5**). Sodium is important for maintaining water balance and neural and muscular functions. Additionally, calcium salts provide skeletal rigidity, whereas calcium ions are involved in many metabolic processes (Freire et al., 2017). Significant decreases in the levels of potassium, manganese, and phosphorus may be explained by their utilization by *L. casei*. LAB under aerotolerance conditions use manganese instead of iron in several processes (Archibald, 1986), which may explain manganese depletion in fermented CW (**Table 5**). Iron, copper, and zinc were detected at low levels in fermented and unfermented CW (**Table 5**), and these essential trace elements are required for physiological growth and antioxidative activity (Frassinetti et al., 2006; Shenkin, 2006). Trace element levels detected in this study were similar to those determined in young CW (Lee et al., 2013; Cappelletti et al., 2015). Therefore, this fermented coconut beverage may represent a good source of micronutrients.

**Table 5:** Concentrations of mineral in coconut water fermented with *L. casei* L4 for 48.

Mineral content (μg/mL)	Fresh CW	Fermented CW
Zinc	0.19 ± 0.02^a^	0.20 ± 0.01^a^
Iron	0.07 ± 0.0^a^	0.06 ± 0.01^a^
Boron	0.71 ± 0.06^a^	0.68 ± 0.07^a^
Copper	0.08 ± 0.01^a^	0.06 ± 0.01^a^
Calcium	119.83 ± 6.18^a^	137.42 ± 2.7^b^
Manganese	3.84 ± 0.26^a^	2.83 ± 0.33^b^
Magnesium	127.3 ± 6.2^a^	119.6 ± 3.6^a^
Potassium	2086 ± 37.4^a^	2014 ± 39.6^a^
Phosphorus	136.2 ± 7.1^a^	122 ± 4.7^b^
Sodium	372 ± 24.6^a^	391 ± 21.6^b^

*Results are presented as mean ± SD (n = 3). Values in a row with unlike superscript letters are significantly different.*

**FIGURE 3:**
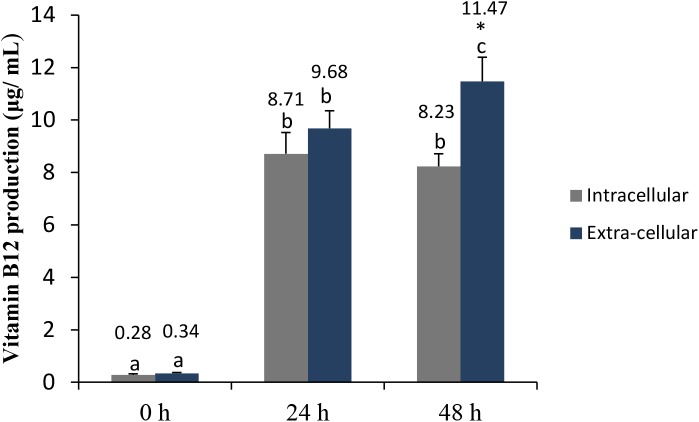
Intra-cellular and extracellular production of vitamin B12 in CW fermented with *L. casei* L4. Different letters above the bar indicate significant differences (*P* < 0.05). Artistic (^∗^) indicate significant different within the treatment.

Vitamin B12, or cobalamin, plays a key role in cellular metabolism and DNA synthesis. It is produced only through microbial synthesis, and animal products represent the principal dietary sources of this vitamin (Herbert, 1994; WHO/FAO, 2004). Here, the intracellular and extracellular vitamin B12 levels produced by *L. casei* L4 were shown to be significantly higher at 24 h than at 0 h (**Figure 3**). However, no differences between the intracellular and extracellular vitamin B12 concentrations were observed at 24 h. Extracellular B12 levels were significantly higher (*p* < 0.05) at 48 h, compared with intracellular B12 levels. Recently, Kantachote et al. (2017) reported optimized conditions that increased extracellular vitamin B12 levels to 14.06 μg/mL in CW after 48 h of fermentation with *L. plantarum* DW12. Therefore, we demonstrated here that the *L. casei* L4 strain can be used for vitamin B12 production as well. In Japan and the United States, the recommended dietary amount of vitamin B12 for adults is 2.5 μg/day (Watanabe, 2007); therefore, *L. casei* L4-fermented CW may represent a beneficial functional beverage for preventing vitamin B12 deficiency, especially in vegetarians. Taken together, these data indicated that CW fermented for 48 h with *L. casei* L4 had good probiotic, antibacterial, and antioxidant activities, maximal vitamin B12 levels, and satisfactory mineral levels.

### Sensory Analysis

Using 20 survey subjects, we evaluated the acceptability of fermented CW (recipe A), fermented CW supplemented with 15% honey (recipe B), and fermented CW supplemented with 15% honey and coconut flavor (recipe C) (**Table 6**). No significant differences in the color or appearance of the samples were observed, but the addition of 15% honey and coconut flavor (recipe C) significantly enhanced the flavor, odor, and overall acceptance of the beverage, indicating that the recipe C was generally acceptable to most of the test subjects. This outcome was most likely due to the decreased acidity and increased sweetness and aroma of this beverage after adding honey and coconut flavor. Our results agree with those reported by Kantachote et al. (2017), which showed that the addition of 20% honey improved the acceptance of MCW fermented with *L. plantarum* DW12. Further, Prado et al. (2015) supplemented fermented CW with sucrose and artificial CW flavor, which improved the acceptance of the investigated beverage. Alternatively, the fermented beverages can be supplemented with fruit ingredients or herbs to improve their appeal to consumers.

**Table 6:** Sensory evaluation of coconut water fermented by *L. casei* L4 and its modified recipes.

Parameter	Recipe A	Recipe B	Recipe C
Color	3.16 ± 0.47^a^	2.94 ± 0.62^a^	3.25 ± 0.68^a^
Appearance	2.85 ± 0.62^a^	2.97 ± 0.45^a^	3.03 ± 0.83^a^
Flavor	2.77 ± 0.75^a^	3.44 ± 0.68^b^	4.26 ± 1.03^c^
Odor	2.65 ± 0.38^a^	2.87 ± 0.44^a^	3.72 ± 0.53^b^
Acceptance	2.92 ± 0.52^a^	3.41 ± 0.57^b^	3.95 ± 0.88^c^

*Recipe A: Fermented CW; Recipe B: Fermented CW plus 15% honey; Recipe C: recipe B plus coconut flavor. Different superscript letter with in the same row indicate significant different (p < 0.05).*

## Conclusion

*Lactobacillus casei* L4, a strain shown to have potential probiotic activities, was demonstrated here to be useful in producing a fermented CW beverage. Following the 48-h fermentation with *L. casei* L4, we determined that the fermented beverage contains essential minerals, vitamin B12, antioxidants, and has antimicrobial activity. Furthermore, even after 28-day storage at 4°C, fermented broth retained the desired *L. casei* L4 levels, which are in accordance with the daily recommended probiotic dose. The fermented CW supplemented with 15% honey and artificial coconut flavor was shown to have the highest acceptance rate by the panelists. Further investigations are required for the development of inexpensive *L. casei* L4-fermented functional beverages with various health benefits.

## Author Contributions

SG designed the work. SG and VS contributed to the experimental analyses. SS helped in the data interpretation, statistical analysis, proofread of the manuscript. VS and SP supervised the work. SG wrote the manuscript. All authors reviewed and contributed to improving the manuscript.

## Conflict of Interest Statement

The authors declare that the research was conducted in the absence of any commercial or financial relationships that could be construed as a potential conflict of interest.
